# Comparison of a tube-holder (Rescuefix) versus tape-tying for minimizing double-lumen tube displacement during lateral positioning in thoracic surgery: A prospective, randomized controlled study

**DOI:** 10.1097/MD.0000000000004486

**Published:** 2016-08-07

**Authors:** Sung Hye Byun, Su Hwang Kang, Jong Hae Kim, Taeha Ryu, Baek Jin Kim, Jin Yong Jung

**Affiliations:** Department of Anesthesiology and Pain Medicine, School of Medicine, Catholic University of Daegu, Daegu, Republic of Korea.

**Keywords:** one-lung ventilation, DLT displacement, double-lumen endotracheal tube, endotracheal tube holder, lateral position

## Abstract

**Background:**

Double-lumen endotracheal tubes (DLTs) are often displaced during change from the supine to the lateral decubitus position. The aim of this study was to determine whether Rescuefix, a recently developed tube-holder device, is more effective than the traditional tape-tying method for tube security during lateral positioning.

**Methods:**

Patients were randomly assigned to a Rescuefix (R) group (n = 22) or a tape (T) group (n = 22). After intubation with a left-sided DLT and adjustment of the appropriate DLT position using a fiberoptic bronchoscope, the DLT was fixed firmly at the side of the mouth by either Rescuefix or Durapore tape. “Tracheal depth” (from the tracheal carina to the elbow connector of the DLT) and “bronchial depth” (from the left bronchial carina to the elbow connector of the DLT) were measured in the supine position using the fiberoptic bronchoscope. After positional change, tracheal and bronchial depths were measured as described above. As the primary endpoint, displacement of the DLT during positional change was evaluated by obtaining the difference in depths measured when the patient was in the supine and lateral decubitus positions. In addition, after lateral positioning of the patient, any requirement for repositioning the DLT was recorded.

**Results:**

After lateral positioning, there were no significant differences in changes in tracheal and bronchial depths between the groups (tracheal depth 6.1 ± 4.4 mm [R group] and 9.1 ± 5.6 mm [T group], *P* = 0.058; bronchial depth 6.5 ± 4.4 mm [R group], and 8.5 ± 4.6 mm [T group], *P* = 0.132). Although the amount of change in tracheal and bronchial depths was not different between the groups, the need to reposition the DLT was significantly lower in the R group than in the T group (32% vs 68%, *P* = 0.016).

**Conclusion:**

This study demonstrated that use of Rescuefix did not reduce the amount of DLT displacement, but it did significantly lower the incidence of DLT repositioning compared with the tape-tying method. Therefore, Rescuefix appears to be an effective alternative to minimizing DLT displacement during lateral positioning in thoracic surgery.

**Trial registration:**

http://cris.nih.go.kr identifier: KCT0001949.

## 1 Introduction

Double-lumen endotracheal tubes (DLTs) are commonly used in thoracic surgery to perform one-lung ventilation, that is, mechanical separation of the 2 lungs. Unfortunately, a change from the supine to lateral decubitus position for thoracic surgery often leads to displacement of the DLT.^[1]^ Displacement of an endotracheal tube (ETT) is known to be the main cause of airway-related complications even when using a single-lumen ETT,^[2]^ and DLT displacement with consequent malposition in particular is more likely to cause severe arterial hypoxemia than other factors such as preoperative condition or intraoperative gas exchange.^[3]^

**Figure 1:**
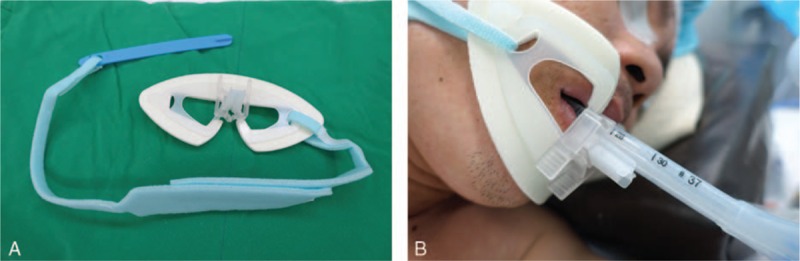
(A) Photograph of the Rescuefix tube-holder device. The plastic tube clamp grips the endotracheal tube. The flexible flange with a foam cushion can adapt to the patient's face. The length-adjustable neck tape wraps the patient's neck with a Velcro strap. (B) Photograph of the Rescuefix device applied to a patient in the lateral decubitus position.

Therefore, securing the ETT safely becomes as important as tracheal intubation itself to prevent displacement of the ETT, and several methods, such as adhesive tape or a tube-holder, have been developed to fix the ETT securely on the patient. Recently, a manikin-based study showed that a tube-holder device provided significantly more tube security than a conventional tape-tying method during simulation of continuous chest compressions.^[4]^ A subsequent clinical study demonstrated that the tube-holder was more effective than adhesive tape in preventing displacement of an ETT in patients undergoing surgery in the prone position.^[5]^ In these studies, the tube-holder device used was the Thomas Endotracheal Tube Holder (Lærdal, Norway), which has a hard plastic face plate and a quick-set screw clamp for fixing the ETT. A more recent development has been the Rescuefix (VBM Medizintechnik, Sulz, Germany), a novel tube-holder consisting of a flexible flange that adapts to the unique shape of an individual patient's face. The Rescuefix also includes a tube clamp that guarantees rapid and safe fixation of the ETT without the use of screw-type devices, so should be able to be applied more easily (Fig. 1). Hence, Rescuefix might be a useful alternative for securing the DLT, even during lateral positioning when displacement of the DLT often occurs. The aim of this study was to determine whether Rescuefix could be more effective than the traditional method of tube fixation using adhesive Durapore tape (3M, St Paul, MN) during lateral positioning in thoracic surgery.

## 2 Methods

### 2.1 Study population

After obtaining approval from the Daegu Catholic University Medical Center Institutional Review Board and informed written consent, patients scheduled for elective thoracic surgery and requiring a left-sided DLT in a lateral decubitus position were enrolled from September 2015 to March 2016. Patients with American Society of Anesthesiologists’ physical status I or II and age 20 to 70 years were included in this prospective, randomized controlled trial. Patients were excluded from participation in the study if they required a right-sided DLT, presented an intraluminal lesion in the left mainstem bronchus, had distorted anatomy of the tracheobronchial tree on chest radiography, or had limited cervical movement. The trial is registered at http://cris.nih.go.kr (KCT0001949).

Eligible patients were randomly assigned in equal numbers to either a Rescuefix (R) group or a tape (T) group in accordance with the method of tube fixation, using random numbers generated by Microsoft Excel 2010 (Microsoft Corp., Redmond, WA), managed by an anesthesia nurse. The patients were not informed of their group allocation, which was concealed in an opaque envelope, managed by an anesthesia nurse who was not involved in the perioperative care, and opened by a staff anesthesiologist (JYJ) immediately before induction of anesthesia.

### 2.2 Anesthetic management and intervention

**Figure 2:**
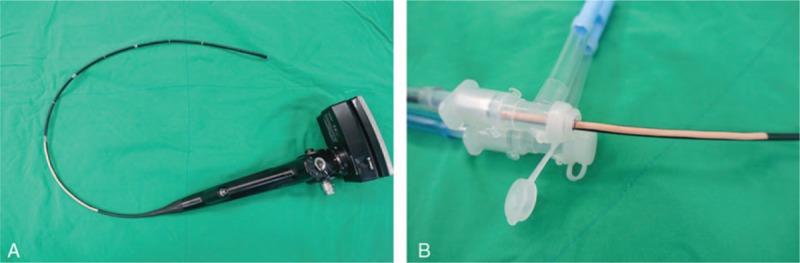
Measurement of depths using the FOB. (A) The tape is attached on the shaft of the FOB approximately 40 cm proximally from the tip. (B) When the tip of the FOB reaches the tracheal or bronchial carina, the point of contact with the elbow connector of the DLT is marked on the shaft of the FOB for measuring “tracheal depth” or “bronchial depth.” Tracheal depth denotes the distance from the tracheal carina to the elbow connector of the DLT. Bronchial depth denotes the distance from the bronchial carina to the elbow connector. DLT = double-lumen endotracheal tube, FOB = fiberoptic bronchoscope.

All patients received midazolam 0.05 mg/kg intramuscularly 30 min prior to induction of anesthesia. Standard monitoring, including an electrocardiogram, a noninvasive blood pressure device, and pulse oximetry, was applied on arrival in the operating room. A disposable bispectral index sensor (BIS, Aspect Medical Systems, Newton, MA) was used to monitor the depth of anesthesia. Anesthesia was induced and maintained with propofol and remifentanil using target-controlled infusion based on bispectral index monitoring of depth of anesthesia, and 0.8 mg/kg of rocuronium was administered for intubation. Tracheal intubation was performed with a disposable polyvinyl chloride left-sided DLT (Broncho-Cath, Mallinckrodt Medical Ltd., Athlone, Ireland); the method used to select the DLT size was based on the reports of Brodsky et al^[6]^ and Hannallah et al^[7]^ using chest computed tomographic scanning. After the bronchial tip of the DLT passed beyond the vocal cords, the stylet was removed, and the DLT was rotated 90° to the left and then advanced until slight resistance was encountered. The position of the DLT was checked and corrected using a fiberoptic bronchoscope (FOB; Olympus Optical Co., Tokyo, Japan) and a blue-colored bronchial cuff was positioned just below the carina without herniation when both cuffs of the DLT were inflated. After identifying the appropriate position for the DLT, an anesthesiologist not involved in the study fixed the DLT firmly to the nondependent side of the patient's mouth using either the Rescuefix or Durapore tape according to group allocation. After fixation of the DLT, “tracheal depth” (from the tracheal carina to the elbow connector of the DLT) and “bronchial depth” (from the bronchial carina to the elbow connector) were measured in the supine position using the FOB. When the tip of the FOB reached the tracheal or bronchial carina, the point of contact with the elbow connector of the DLT was marked on a 15 cm long thin tape attached beforehand on the shaft of the FOB at approximately 40 cm proximally from the tip (Fig. 2). Next, the patient was placed in the lateral decubitus position with an axillary roll placed to the dependent axilla, and the operating table was flexed under the patient's iliac crest. At that time, the patient was carefully repositioned to minimize movement of the head and neck by holding the DLT with one hand while keeping the head and neck neutral with the other hand; all the positioning procedures were undertaken by an independent senior resident. After lateral positioning, tracheal depth and bronchial depth were measured in the lateral decubitus position using another FOB in the manner mentioned above. All the FOB procedures, including confirmation of the correct position of the DLT and measurement of tracheal depth and bronchial depth, were performed by a single investigator (SHB).

### 2.3 Outcome evaluation

The primary endpoint was displacement of the DLT during positional change. This was evaluated by obtaining the difference in value for each depth between the supine and lateral positions. In addition, the investigator recorded whether the DLT needed to be repositioned after lateral positioning due to an inappropriate position, which was defined as herniation of the blue-colored bronchial cuff with poor lung isolation or advancing too deeply into the left main bronchus with obstruction of the left upper lobe orifice, thereby not ensuring a clear view of the left secondary carina via the bronchial lumen. The DLT position was corrected under FOB guidance after the Rescuefix or Durapore tape was separated from the DLT and both tracheal and bronchial cuffs were deflated.

### 2.4 Statistical analysis

Based on a preliminary study, tracheal displacement of the DLT was 5.5 ± 4.6 mm when using the Rescuefix tube-holder and 10.0 ± 5.3 mm when using Durapore tape. Using the independent Student *t* test, we determined that 21 patients would be required in each group with an α error of 5% and a power of 80%. Allowing for a 10% dropout rate, 24 patients were enrolled in each group. The sample size was calculated using G Power 3.1 software (Heinrich-Heine University, Dusseldorf, Germany). Normally distributed data were expressed as the mean ± standard deviation and analyzed by an independent Student *t* test or 1-way analysis of variance. Categorical data were expressed as the number of patients (%) and were analyzed by the chi-squared test or Fisher exact test. All statistical analyses were performed using IBM SPSS Statistics version 19.0.0 (IBM Corp., Armonk, NY). *P* values <0.05 were considered to be statistically significant.

## 3 Results

**Figure 3:**
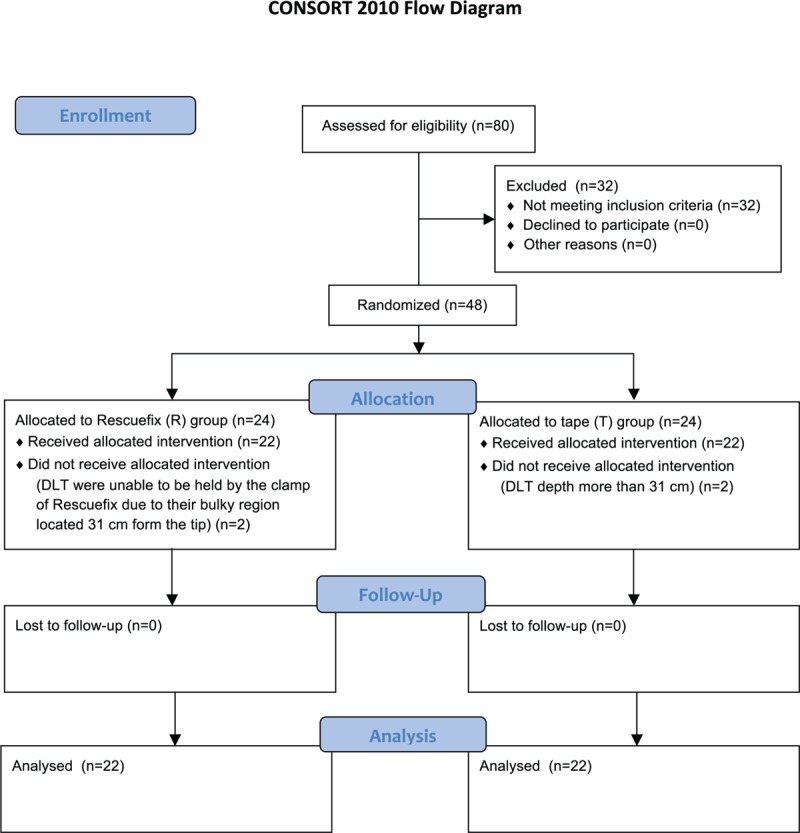
CONSORT flow diagram. DLT = double-lumen endotracheal tube.

Forty-eight of 80 patients scheduled for elective thoracic surgery participated in this study (Fig. 3). Two patients allocated to the R group were excluded because their DLT could not be held by the Rescuefix clamp due to deep placement of the DLT. Durapore tape was used instead of Rescuefix to secure the tube in these 2 patients because the Rescuefix was originally designed for a single-lumen tube and its clamp for tube fixation was somewhat narrow for the bifurcation region located 31 cm from the tip of the DLT. To ensure a balanced comparison, it was planned that patients in either study group whose tube depth was more than 31 cm were to be excluded. Therefore, 2 patients in group T were also excluded from the study. There were no significant demographic differences between the 2 groups (Table 1).

**Table 1:**
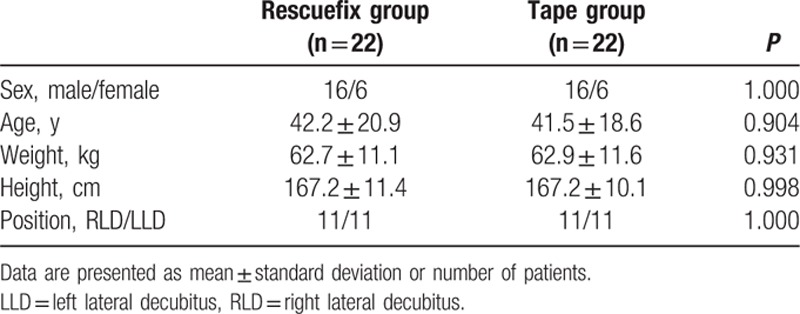
Patient characteristics.

**Figure 4:**
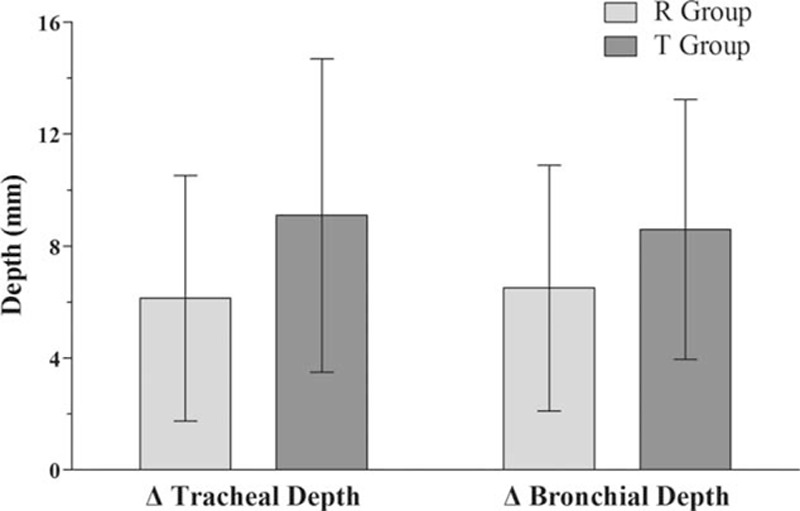
Changes in tracheal depth and bronchial depth during lateral positioning. Data are expressed as the mean ± standard deviation. ^∗^*P* < 0.05 when compared between groups. Tracheal depth denotes the distance from the tracheal carina to the elbow connector of the DLT. Bronchial depth denotes the distance from the bronchial carina to the elbow connector. DLT = double-lumen endotracheal tube, R group = Rescuefix group, T group = tape group.

**Figure 5:**
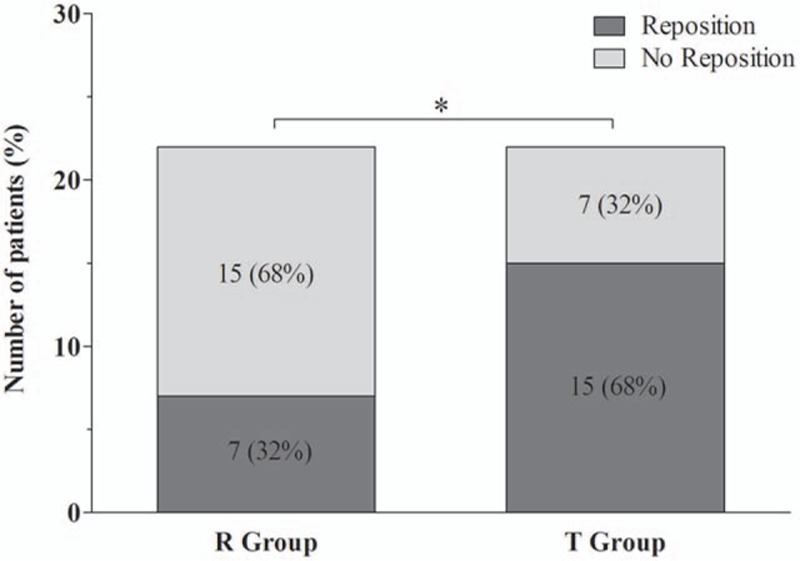
Incidence of DLT repositioning due to inappropriate position. Data are expressed as the number of patients (%). ^∗^*P* < 0.05 when compared between groups. DLT = double-lumen endotracheal tube, R group = Rescuefix group, T group = tape group.

After lateral positioning, displacement of the tracheal lumen and bronchial lumen of the DLT was less in the R group than in the T group (Fig. 4). However, there were no significant differences in changes in tracheal depth and bronchial depth between the 2 groups (change of tracheal depth, 6.1 ± 4.4 mm vs 9.1 ± 5.6 mm, *P* = 0.058; change of bronchial depth, 6.5 ± 4.4 mm vs 8.5 ± 4.6 mm, *P* = 0.132). Despite the lack of a significant difference in the amount of change in these depths, the incidence of actual relocation of the DLT due to inappropriate positioning was significantly lower in the R group than in the T group (32% vs 68%, *P* = 0.016; Fig. 5). However, regardless of tube fixation type, there were no significant differences in changes of tracheal depth and bronchial depth or the incidence of repositioning of the DLT between the right lateral decubitus (RLD) and left lateral decubitus (LLD) position (Table 2).

**Table 2:**
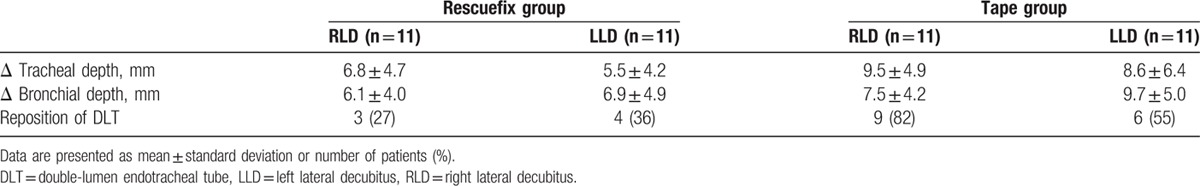
Variables associated with DLT displacement.

Concerning the direction of displacement, both the tracheal and bronchial tips of the DLT moved predominantly upward in both groups. In addition, there was a similar tendency in the direction of malposition in patients whose DLT was repositioned after positional change in both groups (Table 3).

**Table 3:**
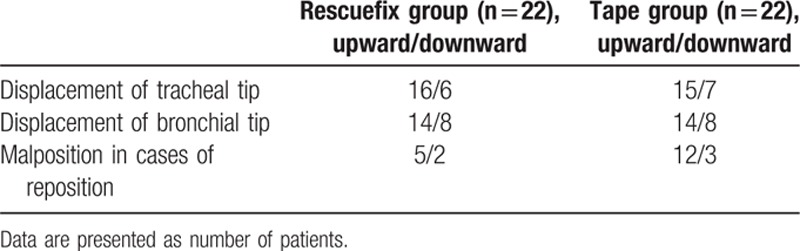
Direction of displacement of double-lumen tube after lateral positioning.

## 4 Discussion

This study demonstrated that displacement of the DLT during lateral positioning was less in patients in whom Rescuefix was used than in patients in whom the traditional tape-tying method was used to secure the DLT, although the difference was not statistically significant. Further, the incidence of repositioning of the DLT owing to an incorrect position was significantly lower among patients in whom Rescuefix was used than in those in whom the tape-tying method was used.

Displacement of the DLT often occurs when the patient's position is changed from supine to lateral decubitus,^[1]^ and consequent malposition of the DLT can lead to failure of one-lung ventilation or even severe arterial hypoxemia during one-lung ventilation.^[2,3]^ In general, patient neck extension has been known to be primarily responsible for such movement of a DLT.^[8]^ Therefore, regarding the method for prevention of DLT displacement, there have been some studies using strategies intended to control head and neck movement, albeit limited in number. Yoon et al^[9]^ demonstrated that restriction of head and neck movement with a neck brace could minimize DLT displacement during lateral positioning, and Seo et al^[8]^ showed that removing a patient's headrest before FOB-guided adjustment of DLT position was an effective strategy for minimizing DLT displacement during lateral positioning. However, an additional mechanism seems to be involved in shifting of the tube, because DLT displacement occurred even in a neutral position of the head and neck in a cadaver model.^[10]^ Desiderio et al^[1]^ mentioned that the dynamics of downward movement of the carina with lateral positioning are known to be related to gravity. Further, a flexed lateral decubitus position may often be required to increase the distance between the costal margins during thoracotomy or to improve exposure of the operative field,^[11]^ and such additional changes in patient position might be responsible for the dynamics of the surrounding organs, including the diaphragm and intra-abdominal organs. In other words, a combination of these factors can affect displacement of the DLT during positional change, and consequently, strategies in addition to restriction of head and neck motion are needed to secure the tube firmly at the level of the mouth to minimize DLT displacement.

As an alternative effective method for securing the ETT, the tube-holder device was evaluated for its usefulness in various situations, albeit not for the DLT. A manikin-based study demonstrated that fixation of the tube-holder significantly reduced the shift in ETT during simulation of continuous chest compression when compared with the tape-tying method.^[4]^ In addition, a study targeting patients undergoing surgery in the prone position showed that the tube-holder device was more effective than adhesive tape in preventing displacement of the ETT.^[5]^ Tube-holders have not been compared with the conventional tape-tying method for preventing displacement of the DLT in thoracic surgery as yet. Therefore, we evaluated the efficacy of the newly developed Rescuefix device, which seems to be easier to apply routinely in patients to secure the DLT during lateral positioning in the operating room than the Thomas Endotracheal Tube Holder used in previous studies.^[4,5]^

In the present study, the Rescuefix could reduce movement of the DLT, thereby decreasing the requirement for repositioning of the DLT when compared with the tape-tying method. Regarding the degree of displacement, Desiderio et al^[1]^ reported that the DLT was moved by approximately 1 cm, predominantly in the upward direction at both tracheal and bronchial sites during lateral positioning. A recent study using cadaver models in which the left mainstem bronchus was directly fixed to the tip of the bronchial lumen of the intubated DLT with forceps showed that the depth of the DLT measured at the right corner of the mouth increased by approximately 0.5 cm after the positional change from supine to both the RLD and LLD.^[10]^ As the result of a cadaver study, the DLT would be moved by about 0.5 cm upward during lateral positioning regardless of direction if the tube was fixed at the mouth. Considering the size of the bronchial cuff of the DLT (about 1 cm) and the range of correct placement of this cuff within the left mainstem bronchus just below the carina, even such modest movement of a DLT (0.5–1.0 cm) should be avoided to achieve one-lung ventilation successfully. In the present study, although the amount of displacement was not significantly different between the 2 groups using either the Rescuefix or tape-tying method, even small movements might induce inappropriate positioning, thereby causing an actual difference in clinical outcomes between these 2 methods. Given the clinical outcome, Rescuefix could actually reduce the incidence of relocation of the DLT and be an effective alternative strategy for preventing displacement of the DLT during lateral positioning.

Although the amounts of movement of DLTs in previous studies^[1,8–10]^ as well as those in our study were slightly different from each other, the predominant direction of the displacement was upward. In other words, the tip of the bronchial lumen of the DLT is likely to be pulled out from the left mainstem bronchus to the trachea during positional change. Therefore, Maruyama et al^[10]^ recommended that the DLT should be inserted approximately 0.5 cm downward from the best position before lateral positioning, and Desiderio et al^[1]^ stated that the bronchial cuff should be at least 1 cm inside the left mainstem bronchus, while an adequate tube position should be always verified using FOB after positional change. However, advancing the DLT more distally without identification of an accurate DLT position by FOB seems not to be desirable. The margin of safety is the length of the tracheobronchial tree over which the DLT may be moved or positioned without obstructing an airway, and the distance from the tracheal carina to the bifurcation on the left-sided bronchus is approximately 4 to 5 cm in length, that is, almost 3 times that of the right-sided bronchus.^[12]^ Although the margin of safety in the left mainstem bronchus is wide enough to place the DLT safely in routine practice, it is possible to obstruct the left upper lobe by the bronchial tip of the DLT, which is intended to be advanced beforehand arbitrarily without verifying the precise placement by FOB, especially in cases moving in a downward direction later. Therefore, in order to prevent malposition and malfunction of a DLT after lateral positioning in thoracic surgery, strategies for minimizing the movement of the DLT during positional change are thought to be more safe and important than the deliberate downward insertion of a DLT in preparation for moving upward.

As a tube-securing device, Rescuefix seems to have some advantages in comparison with other types of tube-holder. In previous studies, the investigators used the Thomas Endotracheal Tube Holder, which consists of a hard plastic face plate and quick-set screw clamp, for fixation of the ETT.^[4,5]^ In contrast, the Rescuefix is composed of more flexible material that adapts to the shape of the patient's face and a simpler tube clamp that allows rapid and safe tube fixation without screw whirling, thereby being easy to apply to patients. Similarly, compared with the conventional tape-tying method, the Rescuefix showed a tendency to facilitate adjustment of the DLT position due to its convenience in manipulation, namely removal and re-application on DLT with the tube clamp. However, the time required to complete these procedures was not statistically tested because there were few cases who needed their tube position rectified. Therefore, further studies with more patients are required in order to determine whether the Rescuefix is actually beneficial in terms of saving the time required to reposition the DLT when compared with the tape-tying method. Another advantage of Rescuefix is the possibility of using it in patients with facial hair as well as those with facial injuries or burns; just as the Thomas Endotracheal Tube Holder, this device is not affected by secretions.^[13]^ In contrast, the adhesive tape may lose its adhesive properties when in contact with facial hair and oral secretions from patients, thereby facilitating tube displacement. Thus, the Rescuefix tube-holder may be a useful device in cases where it is difficult to apply the traditional adhesive tape for tube security, and moreover has benefit as an alternative for patients in whom adhesive tape loses its ability to secure the DLT because of excessive salivation or facial hair.

There are some limitations to this study. First, the investigators could not be blinded to group allocation due to the nature of the interventions, which could be a source of bias. Second, some inaccuracies were present in the measurement of tracheal and bronchial depth. In order to evaluate DLT displacement, these distances between the carina and the DLT lumen were measured conventionally in several studies and the same method was used in the present study. To measure these parameters, our investigator marked the point of contact with the elbow connector of the DLT on the shaft of the FOB when the tip of the FOB reached the tracheal carina or bronchial carina. However, flexion or extension of the tip of the FOB was usually needed to achieve definite contact with the carina, and such movement could introduce some errors when obtaining data associated with the depths. To reduce such errors, a single anesthesiologist who was skilled at manipulation of the FOB was solely responsible for the measurement. However, some degree of error seemed to be unavoidable because of the discrepancy in diameter between the DLT and the FOB. Third, Rescuefix could not be used in patients whose tube depth was more than 31 cm because the DLT bifurcation region is too bulky to fit the tube clamp of the Rescuefix. In fact, there were several tall patients who underwent thoracic surgery because patients with spontaneous pneumothorax tend to have a tall and thin body habitus. Several studies have showed a statistically significant correlation between body height and the optimal insertion depth of a DLT.^[14–16]^ In our study, 2 patients in whom Rescuefix was not feasible were excluded, although some patients who were taller were able to be included in the study. In patients whose tube depth is too deep, there is a risk of DLT kinking through the long route and of movement of the DLT. Therefore, caution is needed when securing the DLT in patients whose tube depth is too deep. Finally, DLT displacement can occur at any time during a surgical procedure, not only during lateral positioning. Unfortunately, we evaluated DLT displacement during lateral positioning only, and did not investigate the subsequent events. Therefore, the results of our study showed the usefulness of Rescuefix during a limited period only. Moreover, considering the high frequency of DLT displacement during the entire period of an operation, the ease of manipulation of tube fixation devices during DLT repositioning as well as the prevention of DLT displacement using a tube fixation device should be considered an important factor. Further studies with extended observation periods are needed to overcome this limitation. If the study period is extended and the ease of manipulation of the device during DLT relocation is assessed, the usefulness of Rescuefix throughout the operation will be able to be evaluated more definitively.

In conclusion, Rescuefix could reduce the amount of DLT displacement during lateral positioning, albeit not to a significant extent when compared to the tape-tying method, and thus could effectively lower the frequency of DLT repositioning after lateral positioning. With this benefit, Rescuefix appears to be an effective alternative to the conventional tape-tying method which minimizing the risk of DLT dislodgement during lateral positioning in thoracic surgery.
